# Risk of retinal artery occlusion in patients with primary open-angle glaucoma: a retrospective cohort study

**DOI:** 10.1186/s40942-026-00836-z

**Published:** 2026-03-23

**Authors:** Nadav Shemesh, Itay Nitzan, Shoham Kubovsky, Tehila Shalmov, Yossi Eshel, Nir Erdinest, Itay Lavy, Jaime Levy

**Affiliations:** Department of Ophthalmology, Hadassah Medical Center, Faculty of Medicine, Hebrew University of Jerusalem, Jerusalem, Israel

**Keywords:** Primary open-angle glaucoma, Retinal artery occlusion, Cohort study, Real-world data

## Abstract

**Background:**

To evaluate the risk of Retinal Artery Occlusion (RAO) in individuals with Primary-Open Angle Glaucoma (POAG) using a large-scale real-world database.

**Methods:**

This retrospective cohort study used the TriNetX Global Collaborative Network to analyze electronic health records from 145 healthcare organizations. Adults aged 18 years or older with POAG were compared with non-POAG controls. Propensity score matching (PSM) was performed 1:1 on 10 baseline characteristics. The primary outcome was incident RAO occurring from one day after the index POAG diagnosis up to 5 years of follow-up. Hazard ratios (HRs) were calculated using Cox proportional hazard models. Kaplan-Meier analysis was conducted to compare RAO-free survival using log-rank tests.

**Results:**

After PSM, there were 260,677 patients in each cohort (50.9% female; mean age 68.2 years). POAG patients had a significantly increased risk of RAO compared with controls (3.46 vs. 1.94 per 10,000; HR 2.16; 95% CI, 1.94–2.42; *p* < .001). The association persisted in sex-stratified and extended follow-up analyses. Anti-glaucoma medications did not significantly alter risk.

**Conclusions:**

This large-scale cohort study identifies POAG as an independently associated risk marker for RAO. These findings highlight the importance of vascular risk assessment in POAG management and highlight the need for increased vigilance for retinal ischemic events in this population.

**Supplementary Information:**

The online version contains supplementary material available at 10.1186/s40942-026-00836-z.

## Background

Retinal artery occlusion (RAO), including central retinal artery occlusion (CRAO) and branch retinal artery occlusion (BRAO), is an ophthalmic emergency with potentially devastating consequences for visual acuity and quality of life. It is associated with significant long-term sequelae, including an elevated risk of subsequent vascular events [1, 2]. Pathophysiologically, RAO results from arterial obstruction due to embolism or compromised ocular perfusion, either from intravascular stenosis or extravascular compression, leading to ischemic damage and retinal cell injury. Known risk factors include carotid artery disease, type 2 diabetes mellitus, and hypertension [1]. 

There is a considerable variability in RAO incidence. CRAO occurs in 1–10 per 100,000 individuals annually, while BRAO is less well characterized. However, asymptomatic RAOs have been reported in up to 1.4% of certain populations, and their incidence increases markedly with age [1, 3]. 

Primary open-angle glaucoma (POAG), the most prevalent form of glaucoma [4], is a chronic, progressive optic neuropathy marked by degeneration of retinal ganglion cells and their axons, resulting in gradual optic nerve atrophy. Although increased intraocular pressure (IOP) is a key factor in most cases, damage may also occur with normal IOP levels [5]. POAG pathogenesis involves impaired aqueous humor drainage, vascular dysregulation, impaired ocular perfusion, and increased susceptibility of retinal ganglion cells to ischemic and oxidative stress [6]. Established risk factors include aging, genetic predisposition and systemic vascular comorbities such as hypertension and diabetes mellitus [7]. POAG is one of the leading causes of irreversible blindness worldwide, affecting more than 50 million individuals in 2020, and its burden is expected to increase [8]. 

Given that POAG alters retinal vascular hemodynamics, either via transmural effects or by impacting venous blood flow [9], it is plausible that a relationship between POAG and RAO may exist. However, evidence supporting this link remains inconclusive. Cohort studies have generally failed to demonstrate an association between glaucoma and RAO [10], while case-control studies have yielded conflicting findings; some report no relationship [11], whereas others suggest a statistically significant association, indicating glaucoma may be a potential risk factor for RAO [12]. These discrepancies may reflect differences in study design, follow-up duration, event rarity, and surveillance intensity, which can substantially influence the detection of a relatively uncommon outcome such as RAO.

The association between POAG and retinal vein occlusion (RVO) is well-established [9], and POAG management is recognized by the American Academy of Ophthalmology as important in RVO prevention [13]. Yet, no such consensus exists regarding RAO. This may partly relate to distinct vascular mechanisms: elevated IOP can mechanically impair venous outflow, predisposing to RVO, whereas RAO is more commonly embolic or thrombotic and may depend more on systemic vascular risk and ocular perfusion dynamics. nonetheless, both conditions involve inner retinal perfusion and may share a background of vascular dysregulation.

This study aims to investigate the potential relationship between POAG and RAO using a large, real-world electronic health record database. Clarifying this association may enhance our understanding of shared pathophysiological mechanisms and inform risk stratification strategies in clinical practice.

## Methods

### Study aim, design, setting and cohort selection

We conducted a retrospective cohort study to evaluate the association between POAG and RAO. Patients were identified using the International Classification of Diseases, Tenth Revision (ICD-10) codes and Anatomical Therapeutic Chemical (ATC) codes (Additional file 1). The POAG cohort included adults (≥ 18 years) with a diagnosis of POAG (ICD-10 H40.11).

To reduce potential follow-up bias, inclusion required at least one documented follow-up visit (ICD-10 Z00.0) at least one year after the index diagnosis. Controls were patients aged ≥ 18 years with no history of POAG (ICD-10 H40.11) and at least one follow-up visit one year after baseline. The index event was defined as the first date on which all inclusion criteria were satisfied.

### Data source

TriNetX is a global federated health research network that provides access to electronic medical records (EMRs) from numerous large healthcare organizations (HCOs). The platform performs rigorous data preprocessing to minimize missingness and standardizes records through a consistent clinical model with uniform semantic definitions. TriNetX data undergo quality checks for conformance, completeness, and plausibility [14]. The network has been externally validated [15] and successfully employed in diverse clinical studies [16–18]. 

For this study, we utilized the TriNetX Global Collaborative Network, which, as of June 2025, comprised 145 HCOs representing over 165 million patients, primarily from the United States and Western Europe. All data are deidentified and compliant with the Health Insurance Portability and Accountability Act (HIPAA), negating the need for informed consent. The research was conducted in accordance with the Declaration of Helsinki. The study was granted exemption from the Hadassah Medical Center Institutional Review Board.

### Baseline characteristics and statistical analysis

Baseline characteristics, including demographics (age, sex, race, ethnicity), comorbidities, and pharmacologic treatment, were extracted from the 5-year window preceding the index date (1,825 days to 1 day prior). A standardized mean difference (SMD) ≥ 0.1 was considered indicative of an imbalance between cohorts.

### Propensity score matching (PSM)

Propensity score matching was conducted using TriNetX’s built-in algorithm, which implements logistic regression with scikit-learn (Python 3.7). Greedy nearest-neighbor matching was used at a 1:1 ratio, with a caliper of 0.1 pooled standard deviations of the propensity score logit. The covariate matrix was randomized prior to matching to prevent ordering bias. No imputation was performed.

Matching was based on 10 variables. Demographic covariates included age at index, sex, and racial/ethnic categories (White, Black or African American, and Asian). Clinical covariates included type 2 diabetes mellitus (ICD-10 E11), carotid artery stenosis (ICD-10 I65.2), neoplasms (ICD-10 C00–D49), essential (primary) hypertension (ICD-10 I10), disorders of lipoprotein metabolism and other lipidemias (ICD-10 E78), tobacco use (ICD-10 Z72.0), and overweight and obesity (ICD-10 E66). Balance was verified visually using propensity score distribution plots (Additional file 2) and numerically in Table 1.

**Table 1:** Baseline characteristics of patients With POAG vs. controls, before and after propensity score matching

		Patients, No (%)					
		Before propensity score matching			After propensity score matching*		
Characteristic	ICD-10 Code	POAG (*N* = 260,682)	Control (*N* = 10,438,167)	SMD	POAG (*N* = 260,677)	Control (*N* = 260,677)	SMD
Age at Index, mean ± SD (years)	NA	68.2 ± 11.4	47.3 ± 17.5	1.399	68.2 ± 11.2	68.2 ± 11.3	0.001
Sex							
Female, No. (%)	NA	129,965 (52.9)	5,805,416 (55.7)	0.095	129,965 (50.9)	129,978 (52.9)	< 0.001
Race							
White, No. (%)	2106-3	128,579 (50.3)	6,842,437 (65.6)	0.312	128,579 (50.9)	128,567 (50.4)	< 0.001
Black or African American, No. (%)	2054-5	66,617 (26.1)	1,282,985 (12.3)	0.356	66,610 (26.1)	66,756 (26.2)	0.001
Asian, No. (%)	2028-9	10,027 (3.9)	395,411 (3.8)	0.193	10,027 (3.9)	10,061 (3.9)	< 0.001
Baseline diagnosis							
Type 2 Diabetes Mellitus, No. (%)	E11	75,907 (29.7)	914,883 (8.8)	0.552	75,900 (29.7)	75,953 (29.8)	< 0.001
Occlusion and Stenosis of Carotid Artery, No. (%)	I65.2	10,353 (4.1)	102,936 (1.5)	0.097	5,365 (2.8)	5,364 (2.8)	< 0.001
Neoplasms, No. (%)	C00-D49	26,128 (13.4)	779,901 (11.7)	0.051	26,109 (13.4)	26,102 (13.4)	0.001
Essential (primary) hypertension (%)	I10	136,786 (53.6)	2,371,715 (22.7)	0.700	136,779 (53.6)	136,876 (53.6)	< 0.001
Disorders of lipoprotein metabolism and other lipidemias, No. (%)	E78	1,901 (0.8)	64,451 (0.6)	0.015	1,901 (0.7)	1,420 (0.6)	0.023
Tobacco use, No. (%)	Z72.0	6,150 (2.4)	235,108 (2.3)	0.010	6,150 (2.4)	6,038 (2.4)	0.003
Overweight and obesity, No. (%)	E66	46,175 (18.1)	1,073,357 (10.3)	0.225	46,170 (18.1)	46,160 (18.1)	< 0.001

Abbreveations: POAG, primary-open angle glaucoma; ICD-10, International Classification of Diseases, Tenth Revision; SMD = Standard Mean Difference; SD, Standard Deviation.* Propensity score matching (PSM) was conducted based on 10 characteristics: age at index, sex, race, type 2 diabetes mellitus, occlusion and stenosis of carotid artery, neoplasms, essential hypertension, disorders of lipoprotein metabolism, tobacco use and overweight/obesity

### Outcomes and follow-up

The primary outcome was a new diagnosis of RAO after the index event, identified using ICD-10 codes for transient (H34.0), central (H34.1), and other (H34.2) RAOs. Patients with a RAO diagnosis prior to the index event were excluded.

Follow-up extended from one-day post-index to the earliest of five years, last EMR entry, or outcome event.

To validate the findings, we included:

- Positive control: Blindness and low vision (ICD-10 H54).
- Negative control: Alcoholic liver disease (ICD-10 K70).

To protect confidentiality, cell sizes between 1 and 9 were rounded up to 10 per TriNetX policy.

### Survival and hazard analyses

Kaplan-Meier survival curves were generated to compare RAO-free survival between POAG and control cohorts, with log-rank testing using the survival package in R (v3.2.3). Hazard ratios (HRs) and 95% confidence intervals (CIs) were estimated using Cox proportional hazards models, with group status as the independent variable.

The proportional hazards assumption was tested using Schoenfeld residuals; violations were flagged at *P* ≤ .01 [19]. 

### Sensitivity and subgroup analyses

To evaluate the robustness of the association between POAG and RAO, we performed:

1. Sex-stratified analyses, comparing males and females with POAG to their matched non-POAG controls.
2. Extended follow-up analyses, repeating the primary analysis with follow-up periods of 10 and 20 years. However, long-term estimates may be influenced by changes in coding practices, healthcare utilization, loss to follow-up, and competing mortality, and data completeness over two decades cannot be fully verified.

### Medication exposure analysis

We assessed the impact of POAG pharmacological treatment on RAO risk within the POAG cohort, comparing patients who received pharmacologic therapy (sympathomimetics, parasympathomimetics, carbonic anhydrase inhibitors, beta-blockers, and prostaglandin analogs) within one month before or after diagnosis were compared to those who did not. PSM was applied to balance treatment-exposed and non-exposed subgroups, and RAO incidence was compared.

## Results

Before PSM, 260,682 patients from 108 healthcare organizations (HCOs) met the inclusion criteria for the POAG cohort, while 10,438,167 patients from 112 HCOs were included in the control cohort. The POAG group patients were older at baseline, with a mean age of 68.2 ± 11.4 years compared to 47.3 ± 17.5 years in the control group (standardized mean difference [SMD] = 1.399).

After PSM, both cohorts included 260,677 patients, with 50.94% and 50.95% females in the POAG and matched control cohorts, respectively. The mean age at index was 68.2 ± 11.2 years in the POAG cohort and 68.2 ± 11.3 years in the matched control cohort, indicating successful demographic matching (Table 1).

**Fig. 1:**
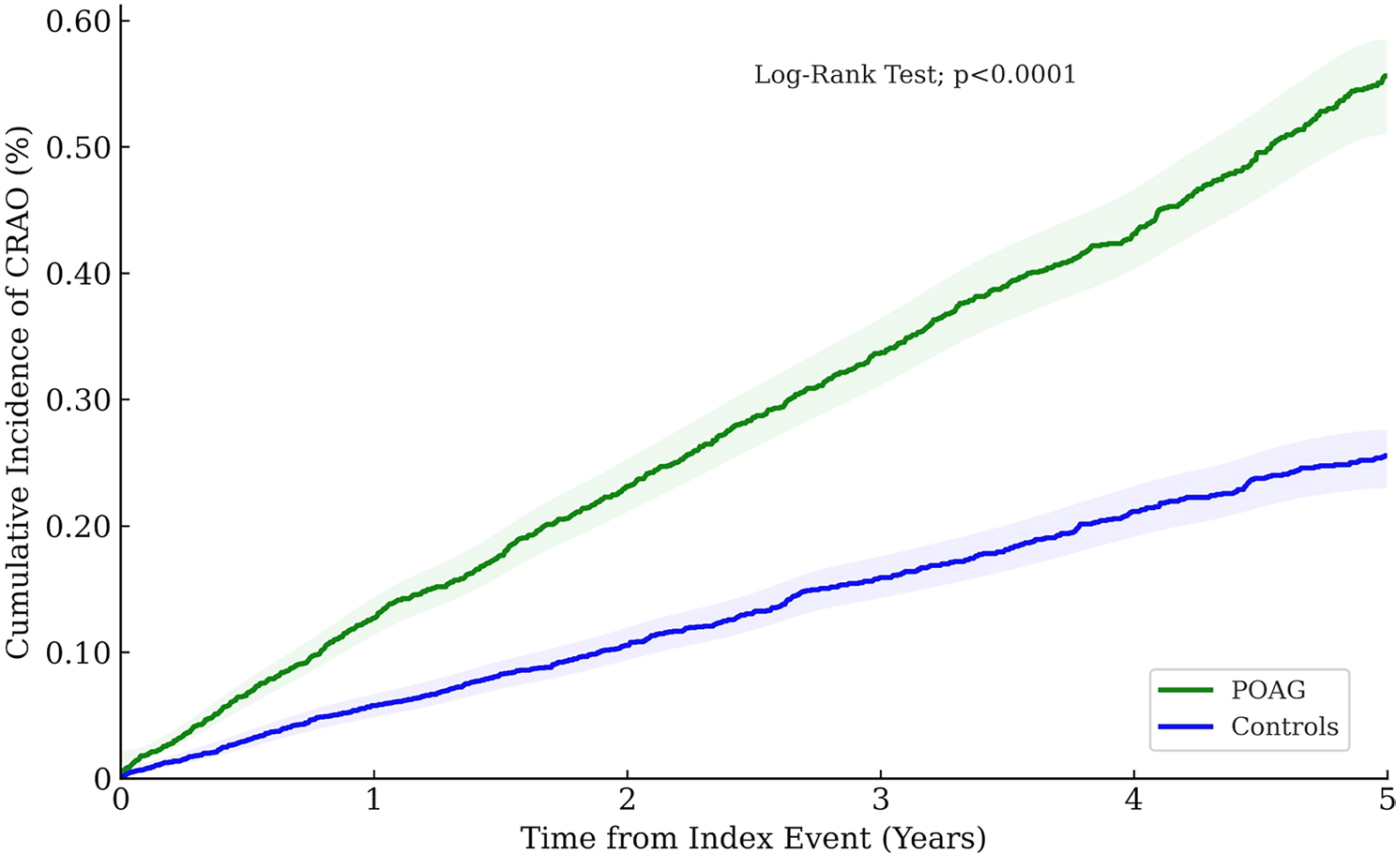
Kaplan-Meier Analysis of POAG and Risk of RAO. This kaplan-meier survival curve illustrates the cumulative incidence of retinal artery occlusion (RAO) over time (in months) in patients with primary open-angle glaucoma (POAG) compared with matched controls. The POAG cohort (green line) exhibited a consistently higher risk of RAO than the control cohort (blue line) throughout the follow-up period; log-rank *p* <.001; HR 2.16; 95% CI, 1.93–2.42

Over the 5-year follow-up period, patients with POAG demonstrated a significantly elevated risk of developing RAO compared to matched controls (3.46 vs. 1.94 cases per 10,000 patients over five years; log-rank *P* <.001). The corresponding hazard ratio (HR) was 2.16 (95% confidence interval [CI], 1.94–2.42) (Fig. 1), reflecting a 116% increase in hazard. The absolute risk difference was 1.52 events per 10,000 patients over five years, corresponding to an approximate number needed to harm of 6,579.

Stratified analyses confirmed the association in both sexes. Among male patients, the HR was 2.21 (95% CI, 1.89–2.58), while among female patients, it was 2.14 (95% CI, 1.82–2.52).

**Fig. 2:**
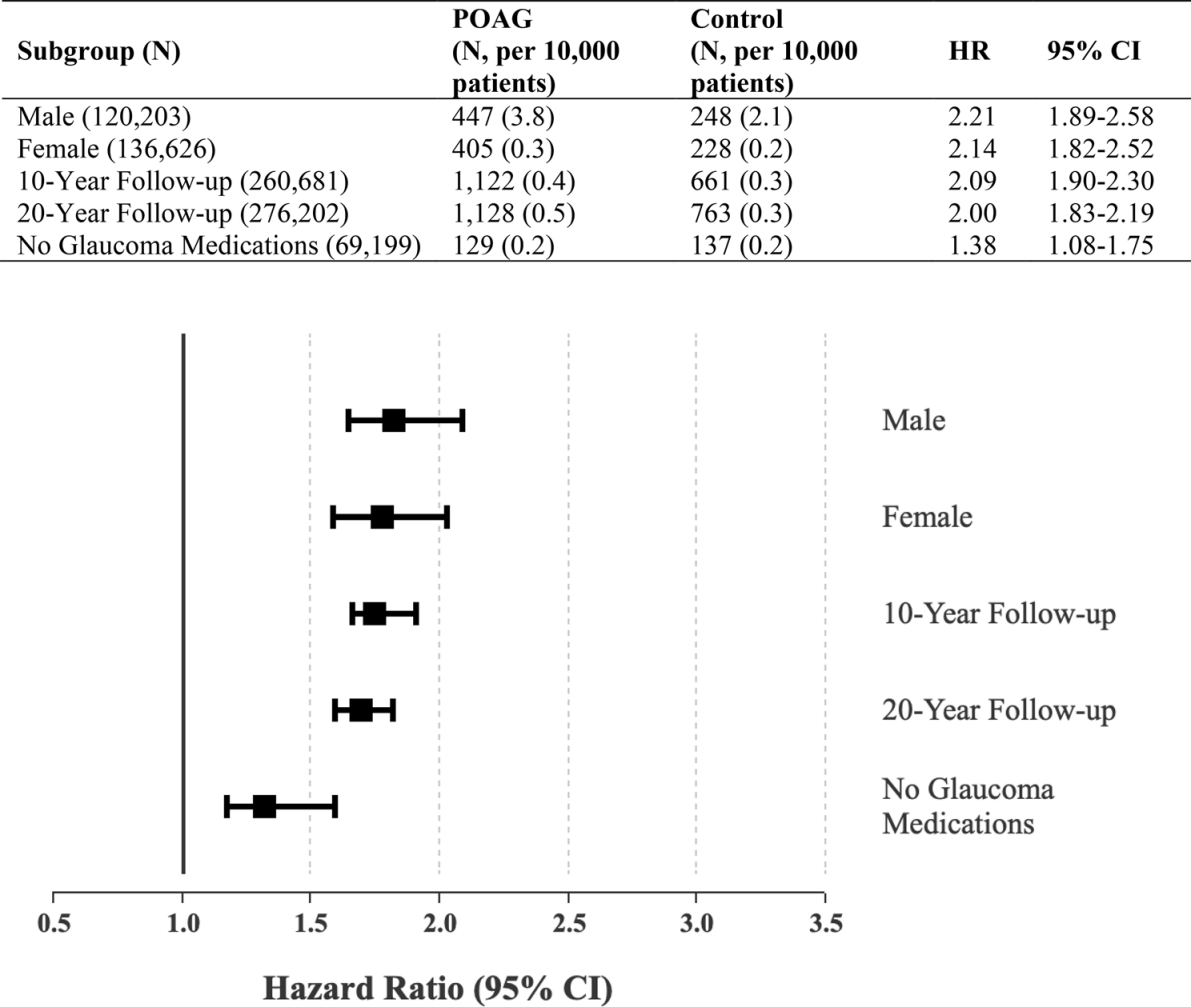
Sensitivity and subgroup analyses for the association between POAG and RAO

Extended follow-up analyses demonstrated sustained risk elevation at 10 years (HR 2.09; 95% CI, 1.90–2.30) and 20 years (HR 2.00; 95% CI, 1.83–2.19) (Fig. 2), although event counts were lower at extended time points and should be interpreted with caution. The 20-year estimates should be interpreted with caution, as long-term follow-up in administrative databases may be affected by changes in coding, healthcare utilization, loss to follow-up, and competing mortality. these results are exploratory and do not represent definitive long-term risk.

In the medication exposure analysis within POAG patients, no significant differences in RAO risk were observed between treated and untreated POAG patients across all major therapeutic classes, including sympathomimetics, parasympathomimetics, carbonic anhydrase inhibitors, beta-blockers, and prostaglandin analogs (Fig. 2).

As expected, the positive control outcome, blindness and low vision (ICD-10 H54), was significantly more frequent in the POAG cohort following PSM. In contrast, the negative control outcome, alcoholic liver disease (ICD-10 K70), showed no significant difference in incidence between POAG and non-POAG patients, supporting the specificity of the primary association.

## Discussion

This large-scale retrospective cohort study demonstrates a significant association between POAG and an increased risk of RAO. Over a five-year follow-up period, POAG patients exhibited a 2.16-fold higher risk of developing RAO compared to matched controls. However, the absolute event rate was low, with a difference of approximately 1.5 cases per 10,000 individuals over five years, which should be considered when interpreting clinical impact. This association remained stable over time and was consistent across sensitivity and subgroup analyses, including sex-stratified cohorts and extended follow-up periods of 10 and 20 years.

To our knowledge, this is the first large epidemiological study to show that POAG is independently associated with an increased risk of RAO. Prior investigations have produced inconsistent findings. In a cohort study, Hayreh et al. analyzed 439 RAO patients (499 eyes) and found no significant difference in the prevalence of neovascular POAG among non-arteritic central RAO (CRAO) cases [10]. Similarly, a case-control study by Schwaber et al. involving 35 RAO patients failed to establish such association [11]. In contrast, a larger case-control study by Ørskov et al. (*n* = 5,312) identified glaucoma as a risk factor, reporting a significantly higher prevalence of prior glaucoma among RAO patients [12]. POAG patients are likely to have more frequent ophthalmologic follow-up than controls, which may increase the probability of rao detection and introduce surveillance bias. although we required at least one follow-up visit in both cohorts and performed propensity matching on major systemic risk factors, differential healthcare utilization cannot be fully excluded as a contributing factor.

POAG and RAO both impact retinal tissue through different pathophysiological mechanisms. POAG is primarily characterized by optic nerve fiber damage, affecting the inner retina, due to elevated intraocular pressure (IOP), with downstream effects on ocular blood flow [20]. This vascular dimension is well-supported by the established link between POAG and retinal vein occlusion (RVO) [9]. Conversely, RAO arises from arterial occlusion, typically of embolic origin, and causes distal ischemia that predominantly affects the inner retinal layers, including the nerve fiber and ganglion cell layers, which are supplied by the central retinal artery [21]. This anatomical overlap with POAG, which also primarily involves the inner retina, may in fact strengthen the biological plausibility of a shared vulnerability to ischemic or vascular dysfunction.

Despite these differences, several mechanisms may underlie the observed association. Rather than IOP level per se, a shared propensity for vascular dysregulation and impaired ocular perfusion may represent a common biological substrate. Reduced ocular blood flow and impaired autoregulation have been described in POAG, even in eyes with statistically normal or well-controlled IOP [22]. Such perfusion instability may increase retinal susceptibility to ischemic injury in the setting of embolic or thrombotic events [23]. Structural changes to the optic nerve head in POAG [24] may alter adjacent vascular architecture, increasing RAO risk, whether through mechanical distortion or impaired neurovascular regulation [25]. additionally, systemic and ocular factors such as vascular endothelial dysfunction, nocturnal hypotension, and altered choroidal circulation have been implicated in glaucoma progression and may contribute to a pro-ischemic environment. myopia, for example, has been associated with altered ocular hemodynamics, although refractive status was not available in the present dataset.

RAO is often referred to as a “retinal stroke”, and its co-occurrence with cerebral ischemic events is well documented [26]. POAG has been linked to increased stroke risk via several mechanisms [27]. Notably, glaucomatous eyes exhibit reduced ocular blood flow and upregulation of hypoxia-related factors such as hypoxia-inducible factor 1-α, indicating systemic vascular dysfunction that may elevate susceptibility to hypoxic events like RAO [28, 29]. 

Carotid artery disease, a well-established risk factor for RAO(1), has also been implicated in POAG pathogenesis through reduced ocular perfusion [30]. Our analysis controlled for carotid stenosis, yet still revealed a significantly elevated RAO risk in the POAG group, suggesting an independent relationship.

Type 2 diabetes represents another shared risk factor. This metabolic disorder contributes to microvascular damage in the optic nerve, potentially accelerating POAG progression [8], while simultaneously promoting atherosclerosis and embolic risk, predisposing to RAO [1, 31, 32]. Importantly, our matched analysis controlled for type 2 diabetes, and the association between POAG and RAO persisted.

Pharmacologic treatment for POAG warrants consideration as well. Some agents, including prostaglandin analogs and alpha-adrenergic agonists, can influence retinal vasculature and have been linked to increased RAO risk [25]. However, by comparing treated and untreated POAG patients directly, our analysis indicate that RAO risk remains elevated regardless of medication status, suggesting that treatment effects alone cannot explain the association.

The relative lack of research on POAG and RAO may be attributed to several factors. First, clinical attention has focused more on POAG’s relationship with RVO, which is more prevalent and better characterized [13]. Second, while both conditions involve the inner retina, prior studies may have overlooked arterial-specific pathophysiology, which could obscure potential links. Finally, the multifactorial and complex vascular effects of POAG [33, 34] may challenge efforts to establish a clear epidemiological connection with RAO.

This study has several limitations. The use of ICD-coded diagnoses introduces the potential for misclassification. As a retrospective analysis based on electronic health records, the study cannot account for disease severity, clinical decision-making, or imaging findings, factors critical for diagnosis and management. While we employed rigorous propensity score matching to control for major confounders, residual bias is still possible. As with all observational database studies, causal inference cannot be established, and unmeasured confounding variables may persist. A particularly relevant limitation is the potential for differential outcome detection: patients with POAG are typically under regular ophthalmologic care, increasing the likelihood of RAO diagnosis compared to controls, who may have limited ophthalmic contact. Additionally, variability in follow-up duration may introduce surveillance bias, potentially affecting the detection of late-onset RAO events.

Nonetheless, this study’s primary strength lies in its use of a large, diverse real-world dataset from a validated global health network, providing both external generalizability and statistical power. Thorough sensitivity analyses and matching techniques enhance internal validity.

In conclusion, our findings provide robust epidemiological evidence that POAG is associated with an increased risk of RAO, likely reflecting shared vascular and hemodynamic mechanisms. Clinically, this suggests that POAG patients may benefit from enhanced monitoring for retinal vascular compromise and more aggressive management of modifiable vascular risk factors. Future studies should explore the mechanistic underpinnings of this association and assess whether preventive strategies can reduce RAO incidence in POAG patients.

## Supplementary Information

Below is the link to the electronic supplementary material.

Supplementary Material 1

Supplementary Material 2

## Data Availability

The data used in this study are available from the TriNetX Global Collaborative Network and are not publicly available due to data-sharing agreements, but are available from the corresponding author on reasonable request.

## Abbreviations

ATC: Anatomical Therapeutic Chemical
BRAO: Branch retinal artery occlusion
CI: Confidence interval
CRAO: Central retinal artery occlusion
EMR: Electronic medical record
HCO: Healthcare organization
HIPAA: Health Insurance Portability and Accountability Act
HR: Hazard ratio
ICD-10: International Classification of Diseases Tenth Revision
IOP: Intraocular pressure
KM: Kaplan–Meier
POAG: Primary open-angle glaucoma
PSM: Propensity score matching
RAO: Retinal artery occlusion
RVO: Retinal vein occlusion
SMD: Standardized mean difference
