# Supplementary material for: Risk of retinal artery occlusion in patients with primary open-angle glaucoma: a retrospective cohort study
Source: Int J Retina Vitreous. 2026 Mar 23;12:69. doi: 10.1186/s40942-026-00836-z (PMC13130781; doi:10.1186/s40942-026-00836-z)
Supplement: Supplementary file 1 — Supplementary Material 1 [file 40942_2026_836_MOESM1_ESM.docx]

**Additional file 1 –** Codes Used for Study Variables

The table outlines the ICD-10 and Anatomical Therapeutic Chemical (ATC) classification system codes utilized in the study. These codes specifically denote the study’s exposures, outcomes, and covariates.

| **Variable** | **ICD-10 Code(s) (Description)** | **ATC Code(s) (Description)** |
| --- | --- | --- |
| Primary-Open Angle Glaucoma (POAG) | H40.11 | — |
| Retinal Artery Occlusion (RAO) | H34.0 (Transient Retinal Artery Occlusion), H34.1 (Central Retinal Artery Occlusion), H34.2 (Other Retinal Artery Occlusion) | — |
| General Medical Examination (Control Cohort Selection) | Z00.0 | — |
| Positive Control Outcome | H54 (Blindness and low vision) | — |
| Negative Control Outcome | K70 (Alcoholic liver disease) | — |
| Matched Diagnosis | E11 (Type 2 diabetes mellitus), I65.2 (Occlusion and stenosis of carotid artery), C00-D49 (Neoplasms), I11 (essential (primary) hypertension), E78 (disorders of lipoprotein metabolism and other lipidemias), Z72.0 (tobacco use), E66 (overweight and obesity) | — |
| Antiglaucoma Medications | — | S01E (ANTIGLAUCOMA PREPARATIONS AND MIOTICS) |
