# Supplementary material for: Risk of retinal artery occlusion in patients with primary open-angle glaucoma: a retrospective cohort study
Source: Int J Retina Vitreous. 2026 Mar 23;12:69. doi: 10.1186/s40942-026-00836-z (PMC13130781; doi:10.1186/s40942-026-00836-z)

**Additional file 2:** propensity score distributions before and after matching

The figure illustrates the density plots for propensity score distributions in Primary-Open Angle Glaucoma (purple) and control (green) cohorts, both before and after propensity score matching (PSM). The left panel shows the distributions prior to matching, highlighting notable disparities between the cohorts. The right panel, on the other hand, presents the post-matching results where the distributions nearly overlap, indicating successful balancing and enhanced comparability between the cohorts.


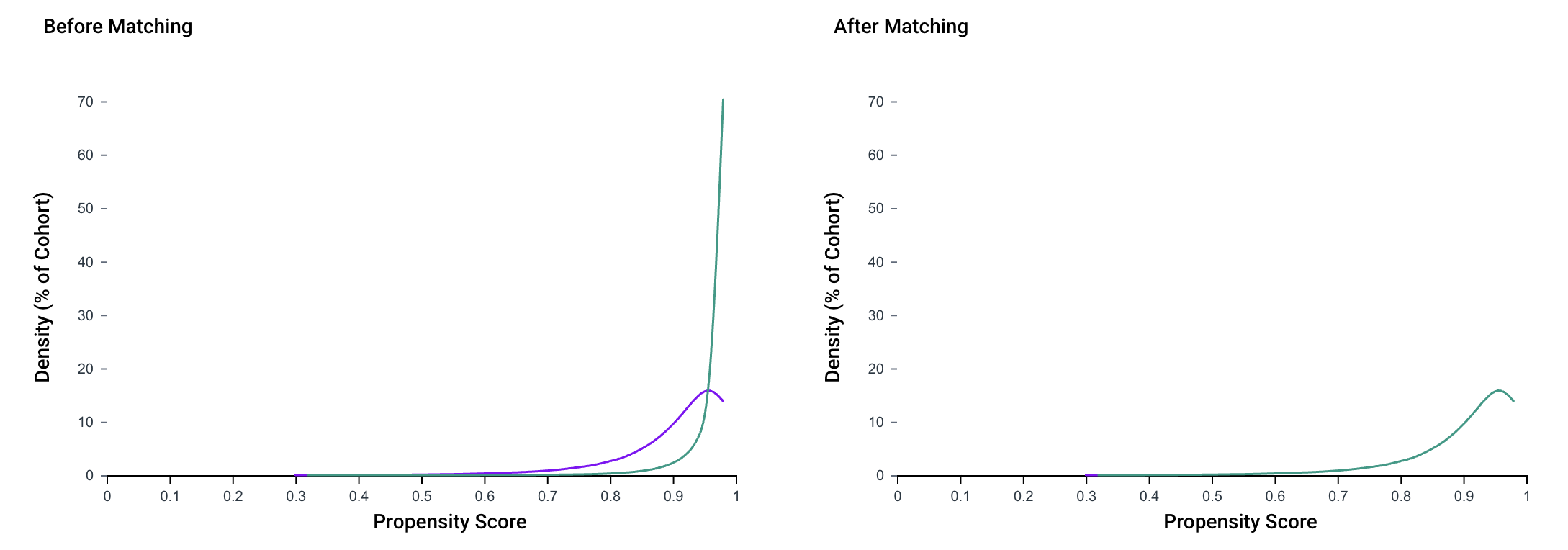

Supplement: Supplementary file 2 — Supplementary Material 2 [file 40942_2026_836_MOESM2_ESM.docx]
